# Nanoscale Electric Characteristics and Oriented Assembly of *Halobacterium salinarum* Membrane Revealed by Electric Force Microscopy

**DOI:** 10.3390/nano6110197

**Published:** 2016-11-02

**Authors:** Denghua Li, Yibing Wang, Huiwen Du, Shiwei Xu, Zhemin Li, Yanlian Yang, Chen Wang

**Affiliations:** 1 Agricultural Information Institute, Chinese Academy of Agricultural Sciences/Key Laboratory of Agricultural Information Service Technology of Ministry of Agriculture, Beijing 100081, China; lidenghua@caas.cn (D.L.); xushiwei@caas.cn (S.X.); lizhemin@caas.cn (Z.L.); 2 CAS Key Laboratory of Standardization and Measurement for Nanotechnology, CAS Center for Excellence in Nanoscience, National Center for Nanoscience and Technology, Beijing 100190, China; ybwang@ecust.edu.cn (Y.W.); duhw@nanoctr.cn (H.D.)

**Keywords:** electrostatic force microscopy (EFM), purple membrane (PM), surface potential, peptides, oriented assembly

## Abstract

Purple membranes (PM) of the bacteria *Halobacterium salinarum* are a unique natural membrane where bacteriorhodopsin (BR) can convert photon energy and pump protons. Elucidating the electronic properties of biomembranes is critical for revealing biological mechanisms and developing new devices. We report here the electric properties of PMs studied by using multi-functional electric force microscopy (EFM) at the nanoscale. The topography, surface potential, and dielectric capacity of PMs were imaged and quantitatively measured in parallel. Two orientations of PMs were identified by EFM because of its high resolution in differentiating electrical characteristics. The extracellular (EC) sides were more negative than the cytoplasmic (CP) side by 8 mV. The direction of potential difference may facilitate movement of protons across the membrane and thus play important roles in proton pumping. Unlike the side-dependent surface potentials observed in PM, the EFM capacitive response was independent of the side and was measured to be at a *dC/dz* value of ~5.25 nF/m. Furthermore, by modification of PM with de novo peptides based on peptide-protein interaction, directional oriented PM assembly on silicon substrate was obtained for technical devices. This work develops a new method for studying membrane nanoelectronics and exploring the bioelectric application at the nanoscale.

## 1. Introduction

Investigations on the electric properties of bio-membranes are vital important to reveal their biological functions and mechanisms. Purple membrane (PM) from the bacteria *Halobacterium salinarum* is a representative protein assembly membrane, which plays a critical role in photochemical energy conversion and protons transporting [1]. PM is a flat 2-D crystalline membrane formed by a hexagonal crystalline lattice of bacteriorhodopsin (BR) trimers in lipids [2]. BR acts as a light-driven, voltage-sensitive proton pump in the PM and serves as an ideal model system to study protein-rich biological membranes at the nanoscale [3]. The structure of BR consists of seven transmembrane α-helices with a chromophore and a photoactive retinal covalently linked to Lys-216 through a protonated Schiff base [4]. The NH_2_-terminal residue, a pyroglutamic acid, is located on the extracellular (EC) surface of the membrane and the COOH-terminal on the cytoplasmic (CP) side. Residues and loops outside the membrane reveal the distribution of charged residues on both sides of the membrane surface. BR converts the energy of single photons into large structural changes to pump protons from the CP side to the EC side across purple membrane directionally, thereby creating an electrochemical gradient used by the ATPases to energize the cellular processes [5]. The function of PM as a light-driven proton pump requires that BR undergoes a cyclic photoreaction, resulting in the release and uptake of protons on the opposite sides of the membrane [6].

On revealing the BR conversion mechanism, a great amount of effort has been made to measure the surface charge density of PM. Charge distribution and charge status of some crucial amino acids on the both sides of the membrane have been determined by high-resolution electron crystallography and the results help provide an insight into how the seven-α-helix membrane protein in BR works [7]. It has been measured that a negative charge density of about 2.5 elementary charges per BR on the CP side and about 1.8 elementary charges per BR on the EC side using the covalently bound pH indicators in aqueous solution [8]. Atomic force microscopy (AFM) is also a powerful tool for studying the structure and the electrical properties of the biomembrane because of its high resolution and multifunctionality. AFM Force curve measurements on the two sides of BR membrane surfaces showed different electrostatic repulsive interactions between the tip and the sample surfaces [9,10]. Many works have reported that the BR crystal surfaces are asymmetric in structural and electrical characteristics due to the asymmetrical distribution of lipids and amino acid residues of BR proteins [11,12,13]. Despite these efforts, it is still difficult to study both the structure and electric properties of biological membranes at the nanoscale. The mechanisms of proton pumping are far from being fully understood, and are thus important for understanding the pumping of membrane protein and for future application of PM.

Electric force microscopy (EFM) is an excellent method to perform two-dimensional electrical characterization with high spatial resolution and minimal cross-talk between topography and potential [14,15,16]. EFM has been extensively used to study surface electric properties in inorganic semiconductors, organic electronics, and biofilms, which were considered as important contributions in the field [17,18,19,20,21]. By introducing external alternating current (AC) and direct current (DC) electric fields, EFM has proven to be a powerful tool in studying structural stability and electric properties of PM membranes at the nanoscale [22,23]. Probing the electric property at the nanometer scale is of fundamental interest because of the rich local structure of the membranes and the fact that many bioelectric phenomena occur at this level [24,25]. The AFM has been introduced to image and quantify the electrostatic properties of protein membranes at (sub-)nanometer resolution in buffer solution, which linked the structure and function relationship of native membrane proteins [26,27]. However, measurements for the electric properties of biomembranes when they are not suspended in an aqueous environment, but rather supported on a suitable substrate in air, are still lacking. It is interesting to characterize the properties of air dried membranes, which may be of importance for some biotechnological applications. In addition, as an important biomaterial with unique electrical properties, BR has been applied in variety of devices [28], such as biosolar cells [29], bioelectronic transistors [30], etc. So the electrical properties of PM on solid surfaces are also crucial for its device applications.

In the current study, we used multi-function EFM to investigate the electric properties of PM. Surface potential and dielectric information of PM patches supported on silicon substrate in air were directly visualized and measured quantitatively. Furthermore, the surface potential differences between the two PM sides were also measured by EFM on peptide-oriented PM at the liquid-solid interface. A peptide showing relatively high binding affinity with the CP side of PM was obtained. The peptide-assisted directional adsorption was further characterized by EFM measurement.

## 2. Results and Discussion

**Figure 1:**
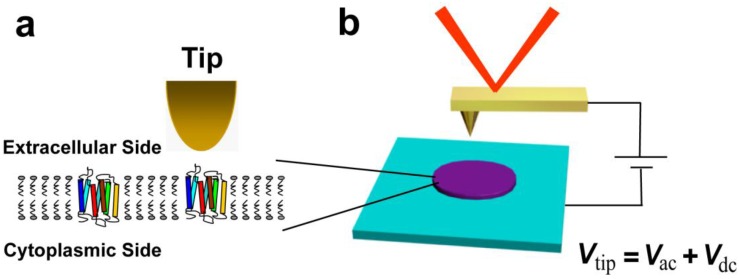
Schematic of (**a**) purple membrane (PM), which consists of bacteriorhodopsin (BR, multicolor) and lipids (gray) only. Oriented PMs are interesting because of their differential electric properties. A BR includes seven-*α*-helices, *C*-terminus, and *N*-terminus; (**b**) Electric force microscope. PMs are deposited on a highly doped silicon substrate randomly oriented face down. Sample topography and electric properties are imaged simultaneously.

The PM samples were deposited on highly doped silicon under ambient condition for the nanocharacterization with EFM measurements. The topographic images and EFM phase were measured on a scanning probe microscopic system (Bruker Dimension Icon, Santa Barbara, CA, USA) with a conductive tip in a two pass tapping mode (Figure 1). By modeling the cantilever as a harmonic oscillator of resonant frequency ω_0_, spring constant *k*, and quality factor *Q*, as well as adopting the standard convention that the measured phase shift Φ = φ + π/2 (where φ is the phase shift between the driving force and the cantilever oscillation), the phase shift over the sample caused by tip-sample capacitive coupling is represented as follows [31,32]:

(1) $$\Phi \left(x\right)=-\frac{Q}{2K}C^{\prime \prime }\left(h\right){\left(V_{tip}-V_{s}\right)}^{2}$$

where *C′′(h)* is the second derivative of the tip-sample capacitance as a function of *h* and *V_s_* is the local electrostatic potential on the sample surface. The phase shift of Equation (1) is zero when *V_tip_* is equal to the value of *V_s_* directly below the tip, so the surface potential can be mapped by EFM.

**Figure 2:**
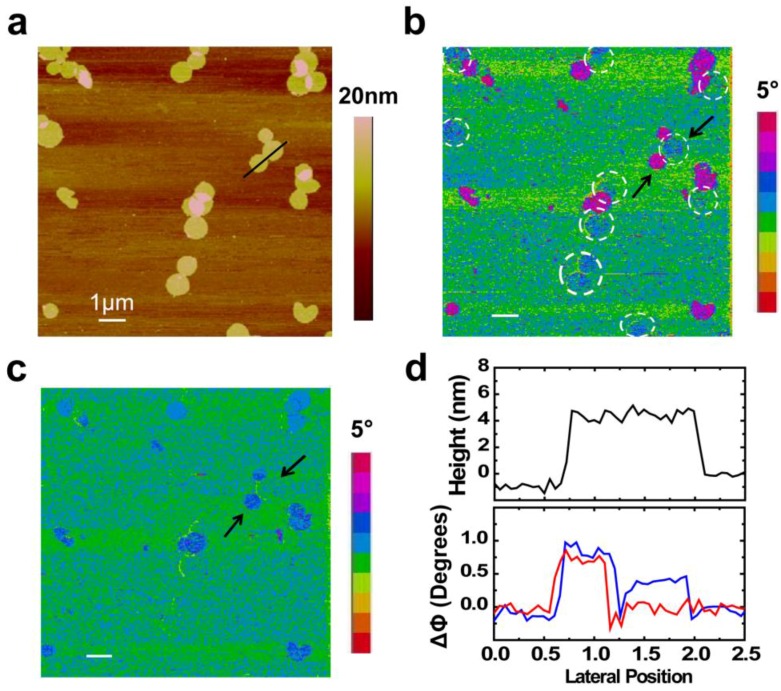
Atomic force microscopy (AFM) and electric force microscopy (EFM) images of PM. (**a**) Topography image of PM patches on silicon; (**b**,**c**) EFM phase images of the sample, with the *V_tip_* = −1 V and 1 V, respectively; (**d**) Line scans of topography and phase centered along the lines in AFM and EFM images. Black curve corresponds to (**a**); blue curve corresponds to (**b**); and red curve corresponds to (**c**). Scale bar in each image is 1 μm.

Figure 2 shows the topography and EFM phase images of the PM prepared on highly doped silicon. The topographic image (Figure 2a) shows that the PM patches are randomly distributed on the silicon, and the fragment is ~500 and ~5 nm in lateral size and thickness, respectively. From the height signal, it is difficult to differentiate the distinction between patches. Figure 2b,c are the corresponding EFM phase images for two different tip voltages. In Figure 2b, a negative voltage (−1 V) is applied on the tip; the phase shift ΔΦ of the PM regions with respect to the bare substrate is positive. As can be seen in Figure 2b, two values of ΔΦ can be identified relative to the silicon substrate, which reflects the difference of EFM phase between the CP side and the EC side of PMs. The counts of the two sides are 14 and 16, nearly 1:1. Patches with deep pink color were more bright and exhibited higher value of phase shift ΔΦ than the others with blue color, as dictated by the bars in Figure 2b. When the tip bias is reversed to a positive voltage of 1 V, the contrast is clearer and the phase shift ΔΦ of patches with white circle is even nearly equal to the bare substrate (Figure 2c). The line scans of topography and phase centered along the lines in AFM and EFM images are shown in Figure 2d. The phase shift ΔΦ between the two sides was about 0.5 degree when the tip was biased 1 V and the phase shift will increase to 1 degree when the tip was biased −1 V. The relative contrast of phase shifts ΔΦ between the CP and EC sides has changed in images taken with tip voltages of opposite polarity, indicative of different values of *V_s_*.

To quantify the surface potential and dielectric property of the EC and CP sides, a phase-locking amplifier was used to detect 1ω and 2ω oscillating signals from the phase shift of the tip. The oscillating electric force at ω acts as a sinusoidal driving force that can excite motion in the cantilever. In regular tapping mode, the cantilever response (oscillating amplitude) is directly proportional to the amplitude of the drive force term. An electrostatic force at the frequency ω and 2ω exerted on the probe is given by [33,34,35]:

(2) $$F_{\omega }=\frac{dC}{dz}abs\left(V_{DC}-V_{CPD}\right)V_{AC}$$

(3) $$F_{2\omega }=\frac{1}{4}\frac{dC}{dz}{V_{AC}}^{2}$$

where *C* and *V_CPD_* are the capacitance and contact potential difference between the sample and tip. Equations (2) and (3) show that the 1ω component of the AFM cantilever deflection signal is related to *dC/dz* and *ΔV_DC_*, whereas the *2*ω component is only related to *dC/dz*, indicating that the *2*ω image is proportional to the dielectric interaction force. *V_DC_* on the tip was adjusted to zero out the contact potential difference (CPD) between the substrate and the probe, until the oscillation amplitude becomes zero and the tip voltage is the same as the surface potential; the derived CPD was used to construct the 2D-surface potential. An amplitude of *2*ω component of the AFM cantilever deflection signal was used to construct the capacitance maps of the same area (Figure S1 of the Supplementary Materials).

**Figure 3:**
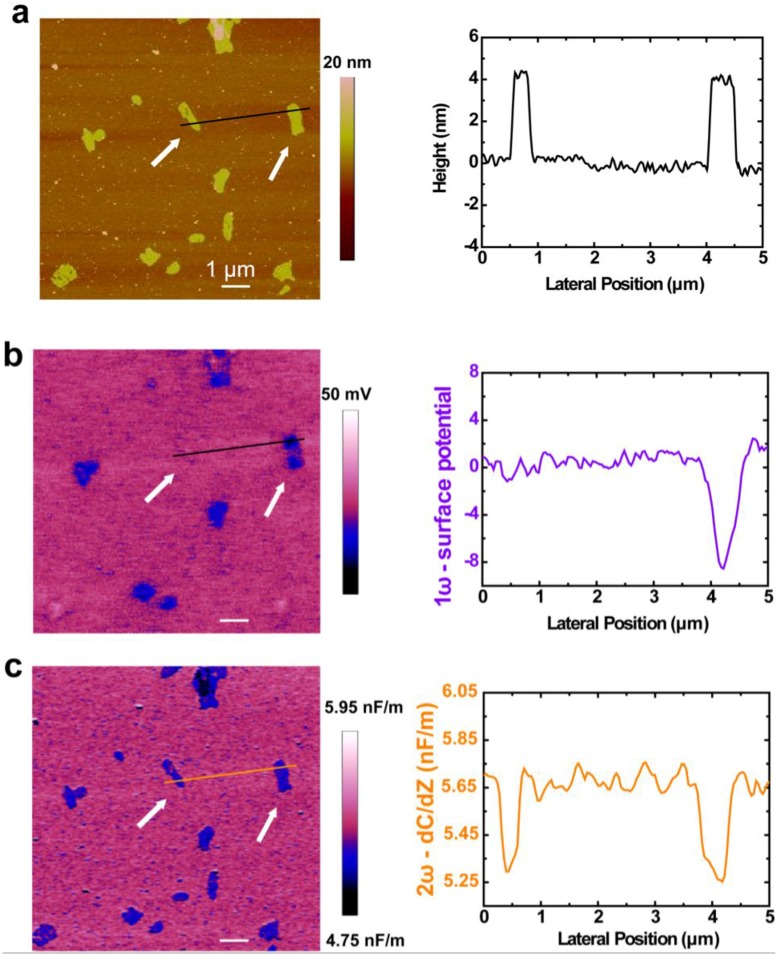
Nanoscale electric imaging of PM on silicon. (**a**) Topography; (**b**) Surface potential image; (**c**) Dielectric capacity derivative image. The line profiles at selected lines as indicated in the images are shown in the corresponding line plots.

Figure 3a shows the topography of the PM samples, a few hundred nanometers wide, deposited on silicon. Figure 3b shows that the surface potential of PM is uniformly distributed within each patch, but varies between the two sides. The membrane is clearly detected by displaying the two surface potential levels of 0 mV and −9 mV relative to the silicon substrate, which reflects the potential difference between the CP side and the EC side of PM. It is known that the potential of the CP side of PM is 8–10 mV higher than that of the EC side [23,36]. Therefore, dark blue patches can be attributed to the EC side facing upward and the lower contrast patches can be attributed to the CP side facing upward. High resolution topography images show the morphological difference between the CP side and the EC side (Figure S2 of the Supplementary Materials). On the extracellular side, four glutamate residues surround the entrance to the proton channel, whereas on the cytoplasmic side, four aspartic acids occur in a plane at the boundary of the hydrophobic–hydrophilic interface. The distinction of amino acid residues between the two sides may give rise to disparate surface charge densities, which could be important to voltage-sensitive proton pump in the PM. As reported previously, the total charge on the CP side of the membrane has a large excess of positive charges, which are themselves surrounded by negatively charged lipids [7]. This configuration may facilitate lateral proton transfer from the lipid area to the entrance of the BR channel. The internal potential difference that crosses the membrane from the CP side to the EC side should be relevant to the proton pump function of BR by attracting protons to be enriched to a high concentration near the CP surface and promoting the motion of charge in the membrane. Experiments on synthetic lipid membranes were done as control experiments to establish that the potential results do arise from PMs but not issues not associated with PMs (Figure S3 of the Supplementary Materials).

Further efforts were made to measure the dielectric property of PM quantitatively. The dielectric property of the membranes is also an important parameter of cell bioelectricity because it quantifies the intrinsic dielectric behavior of the plasma membrane in the low frequency domain (<1 MHz) in the processes, such as membrane potential formation, action potential propagation, or ion membrane transport [37]. EFM has been used as a powerful tool to measure the dielectric property of nanoelectric materials and biomembranes [38,39]. By detecting the amplitude of 2ω component of the AFM cantilever deflection signal using alternating current (ac) detection system, the tip-sample capacitance maps can be constructed in a quantitative way. The capacitance derivative (*dC/dz*) image (Figure 3c) was recorded at a scan height of 20 nm, and clearly detects the membrane by displaying two capacitance derivative (*dC/dz*) levels of ~5.25 and ~5.65 nF/m, which corresponds to the membrane region and the substrate area respectively. The capacitive response of PM is much smaller than that of SiO_2_ (orange line in Figure 3c), indicating that the screening ability of PM to the external electric field is lower than the SiO_2_ substrate. Interestingly, unlike the side-dependent surface potentials observed in PM, the EFM capacitive response is homogeneous and independent of the side. The capacitance levels (*dC/dz*) show an almost homogeneous value for the dielectric constant of the membrane, with a *dC/dz* value of ~5.25 nF/m.

**Figure 4:**
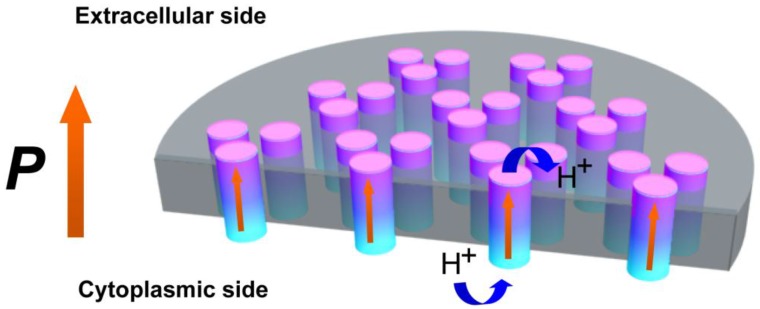
Proposed model of internal structure and potential difference in PM.

Figure 4 shows the proposed model to describe the PM structure and proton transportation. BR trimers in lipids form a hexagonal crystalline lattice arranged into plane. The potential difference of each BR protein crosses the membrane perpendicularly to PM plane in the same direction. The distinction of amino acid residues between the CP side and EC side give rise to disparate surface charge densities, which could form internal potential difference. Since proton pumping of BR was voltage-sensitive, the direction of potential difference from CP side to EC side (pointed out by the orange arrow) may play important roles in pumping protons, energy conversion, and photochromism in the membrane.

PM is exceptionally stable and functional for years when stored in a dried or frozen state. This robustness and easy isolation make the membrane an ideal candidate for optical or electrical devices using its photocycle or charge dislocation properties. Numerous applications can be employed based on its energy conversion and photoelectrism properties [40,41]. Several different approaches have been explored and applied for directional PM assembly in device configurations in the past with varying degrees of success, such as chemical assembly [42], electric field sedimentation [43], and BR mutation [36]. In order to directionally assemble PM on solid substrate and make functional devices, de novo peptides were employed to modify the substrate. Based on protein-protein or peptide-protein interactions, peptide has proven to be a powerful tool in assembly modulation because of its specificity and strong binding affinity [44,45]. In our previous work, peptide targeting at the CP side for oriented PM assembly has been reported, in this work we designed a peptide targeting at EC side for different orientation direction with a sequence of GARGIMIGTGLVGALTDVYSYDF. Binding affinity of peptide with PM was tested by using surface plasmon resonance (SPR), which can provide real-time monitoring of biomolecular interactions [46,47]. The binding constant between PM and the peptide was calculated based on Langmuir adsorption, in which the following equation is applicable.

(4) $$1/R=1/\left(R_{max}K_{A}C_{A}\right)+1/R_{max}$$

*R_max_* is the maximum coverage, *K*_A_ is the equilibrium adsorption constant, and *C_A_* is the concentration of adsorbate solution [48]. The adsorption rate constant of *k_a_* (1/M_s_) was measured to be 283 and the dissociation rate constant of *k_d_* (1/s) was 2.38 × 10^−3^. The equilibrium adsorption constant of *K_A_* was measured to be 1.19 × 10^5^ and the equilibrium dissociation constant of *K*_D_ was 8.43 × 10^−6^. The *K_D_* of the peptide with PM has shown strong binding affinity, which could be chosen as a candidate in assembly modulation.

**Figure 5:**
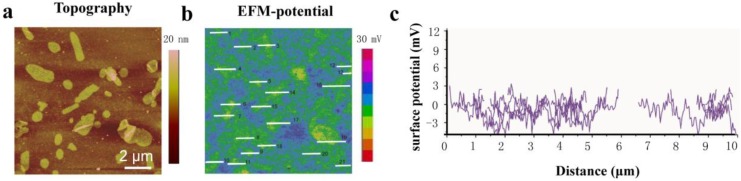
Topography and surface potential of PM on Peptide-3 modified substrates. (**a**) AFM image of PM adsorption on Peptide-3 modified substrates; (**b**) Surface potential image of PM adsorption on Peptide-3 modified substrates; (**c**) Sectional analysis of each patch labeled in EFM potential image. The potentials analysis only gives out one population, ~1 mV, which indicates identical orientation of the PM on surface.

The peptide was modified on silicon substrates via 1-ethyl-3-(3-(dimethylamino)propyl) carbodiimide (EDC)/*N*-hydroxysuccinimide(NHS) coupling for an oriented PM assembly. The silicon surface is treated with oxygen plasma first for hydroxyl group generation and then followed by (3-aminopropyl)triethoxysilane (APTES) modification for an amino-terminated surface. Then, introduction of succine anhydride leads to a carboxyl-terminated self-assembled monolayer (SAM) on the silicon surface, followed by EDC/NHS activation to immobilized peptides onto the silicon surface. Figure 5 shows the topography and surface potential of PM assembly characteristics on peptide modified substrates. The topography image of PM adsorption on silicon substrates shows plenty of PM patches, which could be attributed to the strong interaction between peptides and PM. This observation is consistent with the SPR measurements on the binding affinity of peptide with PM. The surface potential analyses of PM patches on silicon substrate are labeled by lines in EFM-potential images and their sectional analyses were done. Different from no-peptide modified surfaces that have two potential distributions (Figure 3), only one value of potential around 1 mV can be identified relative to the modified silicon surface, which can be attributed to the CP side facing upward. The surface potential measurements clearly show evidence of directional assembly of PM on the silicon surface, which is the key element for potential devices like optical data storage and processing, biosolar devices, ultrafast light detection, and technical biosensors. It is interesting to note that the macroscopic oriented PM devices can be further used for seawater desalination or even exploitation of sunlight in adenosine 5′-triphospate (ATP) generation.

## 3. Materials and Methods

### 3.1. Materials

The culture of *Halobacterium halobium* and PM isolation were carried out following a standard procedure [49]. Purified PM was suspended in double-distilled water and stored at 4 °C. The buffer used in the experiments consisted of 10 mM Tris HCl with a pH value of 8.0.

The peptide with a sequence of NH_2_-GARGIMIGTGLVGALTDVYSYDF-COOH used in the experiments was purchased from Shanghai Science Peptide Biological Technology Co., Ltd. (Shanghai, China).

(3-Aminopropyl)triethoxysilane (APTES), 1-ethyl-3-(3-(dimethylamino)propyl) carbodiimide (EDC), and *N*-hydroxysuccinimide(NHS) were all purchased from Sigma-Aldrich (Darmstadt, Germany) and used without further purification.

### 3.2. Nano-Characterization of Purple Membranes with Electric Force Microscopy Measurements

The PM samples were deposited on highly doped silicon under ambient conditions for the EFM measurements. The topographic images and EFM phase were measured on a scanning probe microscopic system (Bruker Dimension Icon, Santa Barbara, CA, USA) with a conductive tip in a two pass tapping mode (Figure 1). For each scan line, topographic information was obtained in the first pass, and then the tip was lifted to a given constant height of 20 nm above the sample surface and biased a DC voltage *V_tip_* in the second pass. The cantilever was mechanically driven on resonance, and the phase shift of the cantilever oscillation was measured as a function of the tip position. Conducting Pr/Ir coated silicon tips (SCM-PIT, Bruker, Santa Barbara, CA, USA) with a resonance frequency of about 70 kHz were used; the spring constant of the probe was calibrated to be 4.2 N/m in the imaging.

### 3.3. Electric Potential and Dielectric Properties Measurements

The topographic and surface potential images were obtained using a Bruker Dimension Icon microscope in a two-pass operation mode slightly different from the EFM measurement mentioned above. In the first pass, topographic information was obtained. In the second pass, the scan line was the same, followed with the AFM tip 20 nm above the topographical baseline by applying a *DC* bias (*V_dc_*) and a modulating sinusoidal potential *V_ac_* sin(ω*t*) at amplitude of 1 V between the tip and the highly doping silicon substrate, where ω was set to the resonant frequency of the probe. The deflection amplitude of the probe in lift mode was monitored, which was linearly proportional to the electrical force imposed on the probe. *V_dc_* was adjusted to null the contact potential difference (CPD) between the substrate and the probe. The derived CPD was used to construct the 2D-surface potential (SP). A phase-locking amplifier was used to extract the 2ω vibrating signal of the tip; an amplitude of 2ω was used to construct the capacitance maps of the same area. This technique is similar to a combined mode of Kelvin probe force microscopy and scanning dielectric force microscopy. The lateral resolution of our EFM experiment was estimated by (*R* × *z*)^−^^1/2^, which is in the order of several nanometers for the tip radius *R*~20 nm.

### 3.4. Surface Plasma Resonance (SPR) Experiments

The characterization of the peptides-PM binding interaction was accomplished with the surface plasmon resonance technique (KxV5-type SPR, Plexera, Woodinville, WA, USA). This is a highly sensitive method to measure an intermolecular interaction. The peptides were introduced to the surface of the gold chip in advance as stationary phase. SPR chip was modified by thiol molecules containing carboxyl to form self-assembled layer. 5 mL of carboxyl thiols (dithiosole-COOH, 1 μM) and hydroxy-dithiol molecule (dithiosole-OH, 10 μM) mixing ethanol solution was dropped on a SPR chip surface, and then the remaining liquid was removed by suction after 15 min of adsorption. After rinsing with ethanol, the carboxyl-terminated SAM on the chip surface underwent EDC/NHS activation. PM patches were introduced as a mobile phase. Based on the Langmuir adsorption model, the kinetic parameters and the binding constant were calculated using Plexera software.

## 4. Conclusions

In summary, we investigated the electric properties of an extremophile membrane by using multi-function EFM at the nanoscale and demonstrated the ability of EFM to obtain the intrinsic electric potential and low-frequency dielectric capacity of biomembranes. Our results revealed that the PM exhibits asymmetry between the two sides of the membrane under the presence of electric field. The surface potential result shows that the EC sides were more negative than the CP sides by 8 mV. Unlike the side-dependent surface potential observed in PM, the EFM low frequency capacitive response was independent of the side. The dielectric constant of the PM was measured to be at a *dC/dz* value of ~5.25 nF/m. The direction of electric potential difference from CP side to EC side may play important roles in pumping protons and this may be a general mechanism for many ion pump membranes. Directional assembly of surface oriented PM on silicon substrate was obtained, which is the key element for technic devices. These results provide a powerful framework for studying membrane nanoelectronics by which bioelectric devices can be further investigated.

## Supplementary Materials

The following are available online at http://www.mdpi.com/2079-4991/6/11/197/s1, Figure S1: Schematic illustration of muti-function electric force microscopy, Figure S2: High resolution topography images of purple membrane sample prepared on mica and imaged in buffer solution. Figure S3: Surface potential measurements of phosphatidylcholines on silicon.
