# Supplementary material for: Nanoscale Electric Characteristics and Oriented Assembly of Halobacterium salinarum Membrane Revealed by Electric Force Microscopy
Source: Nanomaterials (Basel). 2016 Nov 2;6(11):197. doi: 10.3390/nano6110197 (PMC5245739; doi:10.3390/nano6110197)
Supplement: Supplementary file 1 [file nanomaterials-06-00197-s001.pdf]

Supplementary Materials: Nanoscale Electric Characteristics and Oriented Assembly of *Halobacterium salinarum* Membrane Revealed by Electric Force Microscopy

Denghua Li, Yibing Wang, Huiwen Du, Shiwei Xu, Zhemin Li, Yanlian Yang and Chen Wang

Muti-Function Electric Force Microscopy

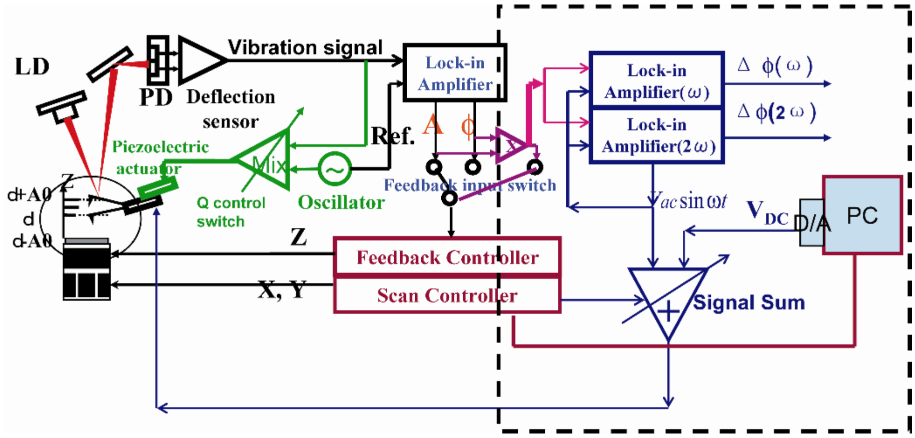

Figure S1. Schematic illustration of muti-function electric force microscopy. The dashed box shows improved alternating current extracting circuit, where the electric potential and capacity can be obtained.

The energy in a parallel plate capacitor is:

$$U = \frac{1}{2}C(\Delta V)^2 \quad \text{(S1)}$$

where C is the local capacitance between the AFM tip and the sample and ΔV is the voltage difference between the two. The force on the tip and sample is the rate of change of the energy with separation distance:

$$F = -\frac{dU}{dz} = -\frac{1}{2}\frac{dC}{dz}(\Delta V)^2 \quad \text{(S2)}$$

The voltage difference, ΔV, in Surface Potential operation consists of both a DC and an AC component. The AC component is applied from the oscillator, V_{AC} sinωt, where ω is the resonant frequency of the cantilever.

$$\Delta V = \Delta V_{DC} + V_{AC}\sin\omega t \quad \text{(S3)}$$

ΔV_{DC} includes applied DC voltages (from the feedback loop), work function differences, surface charge effects, etc. Squaring ΔV produces:

$$F = \underbrace{-\frac{1}{2}\frac{dC}{dz}(\Delta V_{DC}^2 + \frac{1}{2}V_{AC}^2)}_{DCterm} \underbrace{-\frac{dC}{dz}\Delta V_{DC}V_{AC}\sin\omega t}_{\omega term} \underbrace{+\frac{1}{4}\frac{dC}{dz}V_{AC}^2\cos(2\omega t)}_{2\omega term} \quad \text{(S4)}$$

Sub-molecure Resolution Imaging of Two Types of PM Surface Topography Observed in Tapping-Mode AFM. PM samples were diluted to 1 μg/μL in buffers with KCl concentration of 1 M/L. A 10 μL aliquot of PM suspension was pipetted onto a freshly cleaved mica surface and left in air for 10 min to allow absorption. The samples were gently rinsed with imaging buffer (2 mL) and then mounted under the microscope. 20 μL of the same buffer was injected, and the equipment was allowed to stand for 30 min to reach thermal equilibrium. AFM experiments were performed with a commercial microscope (NanoScope IIIa Multi-Mode AFM, Bruker Instruments, Santa Barbara, CA, USA) with a 13 μm scanner and a liquid cell. All of PM topography images were obtained with tapping mode. Due to the nature of acoustic tapping mode in liquid, multiple resonant peaks were observed during "cantilever tune". A drive frequency at a peak around 7 kHz, which is the closest to the cantilever resonance, was selected. Drive amplitude was adjusted so that the tip oscillation amplitude was 0.5 V. Setpoint was finely tuned so that the tip-sample interaction can be appropriately controlled (usually 0.34). The Gains were set to the highest value but do not cause oscillation of tip (usually 0.2). Imaging was carried out with Olympus silicon nitride cantilevers (nominal spring constant *kn* = 0.57 N·m^{-1} (OMCL-TR400PSA-1), Olympus, Tokyo, Japan). Before imaging, the system was left for 2–3 h scanning a blank sample to reach equilibrium. The scan speed was typically 4–9 lines/s for high-resolution frames.

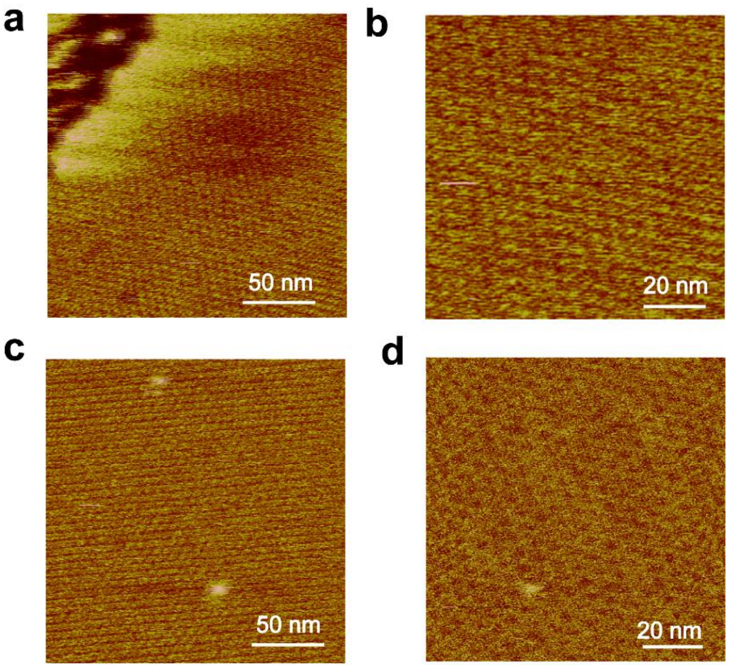

Figure S2. High resolution topography images of purple membrane sample prepared on mica and imaged in buffer solution. (**a**,**b**) 250 nm × 250 nm image and 100 × 100 nm image. The hexagonal lattice of doughnut-shaped monomers with a repeat of 6.4 nm that can be attributed to the cytoplasmic side; (**c**,**d**) 250 nm × 250 nm image and 100 × 100 nm image. The hexagonal lattice of dot-like monomers with a repeat of 6.2 nm that can be attributed to the extracellular side.

It is known that the BR crystal surfaces are structurally asymmetric. One of the membrane surfaces contacts the extracellular solution, the other contacts the cytoplasm. The former surface has a cracked morphology, and the latter evinces a more pitted topography. Both of these surfaces have been investigated previously. In order to identify the orientation at the surface of PM we investigate the topography distinct on both cytoplasmic and extracellular surfaces using high-resolution AM-AFM imaging taken in buffer solution. Figure S1a,b show

high resolution image on cytoplasmic surface, clearly showing the hexagonal lattice of doughnut-shaped monomers with a repeat of 6.4 nm. Figure S2c,d show high resolution image on extracellular surface, clearly showing the hexagonal lattice of dot-like monomers with a repeat of 6.2 nm. The excellent high-resolution images assign the rougher doughnut unit side and the smoother three-dot unit side to the cytoplasmic and extracellular side respectively.

Surface potential measurements of phosphatidylcholines on silicon. Experiments on synthetic lipid membranes were done as control experiments to establish that the potential results do not arise from issues not associated with PMs. Two kinds of typical phosphatidylcholines were used in the experiments: 1,2-Dipalmitoyl-sn-glycero-3-phosphocholine (DPPC) and 1,2-dioleoyl-sn-glycero-3-phosphocholine (DOPC). The phosphatidylcholine samples were deposited on highly doped silicon for the surface potential measurements and the measuring methods were the same with the PMs. The results are as shown below (Figure S3). Figure S3a–d correspond to measurements on DPPC while Figure S3e–f correspond to measurements on DOPC. For DPPC and DOPC lipid, the surface potentials were clearly detected of about −30 mV and −25 mV relative to the silicon substrate respectively, which reflects the lipids were relatively negatively charged. The surface potentials of lipids were demonstrated to be in the same charge ranges as PM but even lower. As for PMs, Figure 3 in manuscript shows that the surface potential of CP side and EC side levels was 0 mV and −9 mV relative to the silicon substrate, which reflects the potential difference between the CP and the EC side of PM. On the CP side of bacteriorhodopsin, negatively charged residues are encircled by positively charged protein residues, which are in turn surrounded by negatively charged lipids and may accumulate protons on the surface.

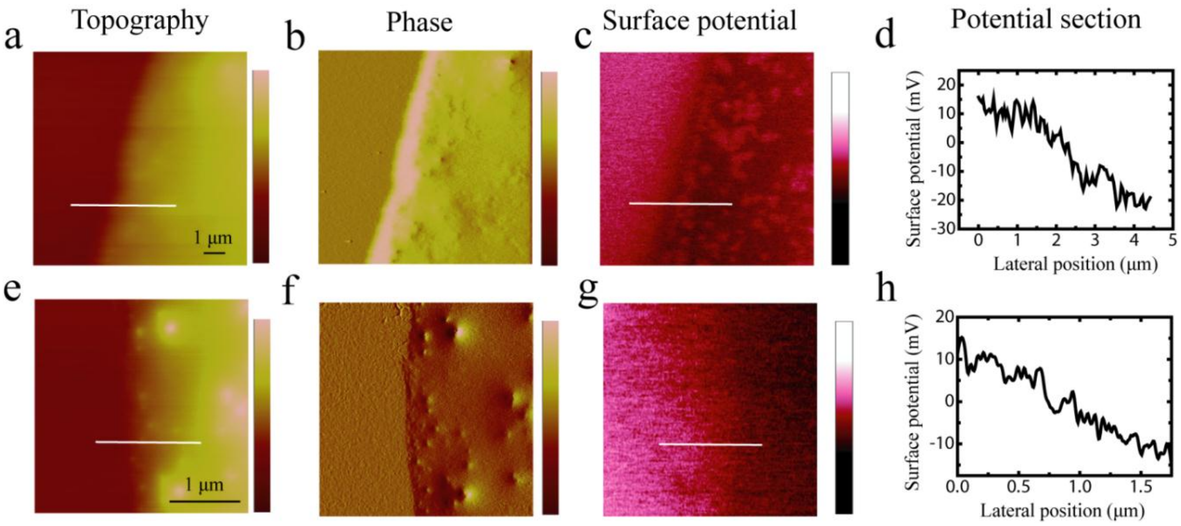

Figure S3. Surface potential measurements of phosphatidylcholines on silicon. (**a**–**d**) correspond to measurements on DPPC while (**e**–**f**) correspond to measurements on DOPC; (**a**,**e**) Topography; (**b**,**f**) Phase image; (**c**,**g**) Surface potential image; (**d**,**h**) Surface potential section at selected lines as indicated in the images.

© 2016 by the authors. Submitted for possible open access publication under the terms and conditions of the Creative Commons Attribution (CC-BY) license (http://creativecommons.org/licenses/by/4.0/).
